# Supplementary material for: Why not to pick your nose: Association between nose picking and SARS-CoV-2 incidence, a cohort study in hospital health care workers
Source: PLoS One. 2023 Aug 2;18(8):e0288352. doi: 10.1371/journal.pone.0288352 (PMC10395815; doi:10.1371/journal.pone.0288352)
Supplement: S1 File — (DOCX) [file pone.0288352.s001.docx]

**S methods.**

1. Do you ever wear glasses?
   - Never
   - Monthly
   - Weakly
   - Daily
2. Did you have a beard during the first and second ‘wave’ of the pandemic?
   - Never
   - Monthly
   - Weakly
   - Daily
3. Do you ever pick your nose?
   - Never
   - Monthly
   - Weakly
   - Daily
   - Every hour
4. Do you ever bite your nails?
   - Never
   - Monthly
   - Weakly
   - Daily
   - Every hour
