# Supplementary figures and images for: Why not to pick your nose: Association between nose picking and SARS-CoV-2 incidence, a cohort study in hospital health care workers
Source: PLoS One. 2023 Aug 2;18(8):e0288352. doi: 10.1371/journal.pone.0288352 (PMC10395815; doi:10.1371/journal.pone.0288352)

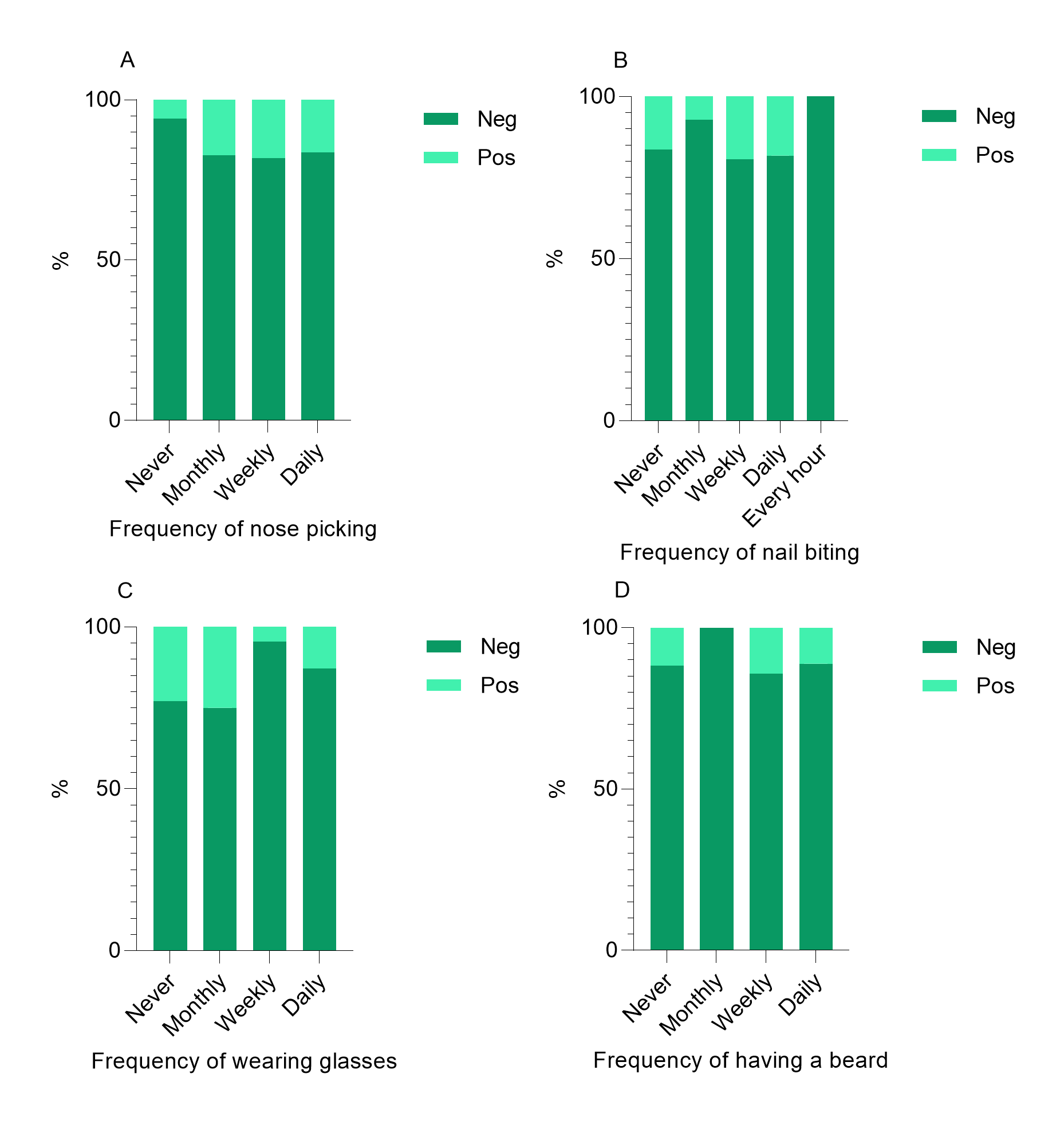

Supplement: S1 Fig — A-D. Bar charts showing the proportion of nose picking, nail biting, and wearing glasses or having a beard in SARS-CoV-2 seropositive and seronegative participants. (TIF) [file pone.0288352.s002.tif]
