# Supplementary material for: Why not to pick your nose: Association between nose picking and SARS-CoV-2 incidence, a cohort study in hospital health care workers
Source: PLoS One. 2023 Aug 2;18(8):e0288352. doi: 10.1371/journal.pone.0288352 (PMC10395815; doi:10.1371/journal.pone.0288352)
Supplement: S1 Table — Baseline characteristics of participants (n = 219) divided into SARS-CoV-2 seropositive and seronegative subgroups. *Males only. (DOCX) [file pone.0288352.s003.docx]

**S1 Table.**

|  | **SARS-CoV-2 seropositive (n=34)** | **SARS-CoV-2 seronegative (n=185)** |
| --- | --- | --- |
| **Working with COVID-19 patients, n (%)** | **23 (67.6%)** | **87 (47.0%)** |
| **-** Emergency department | 4 (11.8%) | 21 (11.4%) |
| **-** Intensive care unit | 5 (14.7%) | 42 (22.7%) |
| - Nursing ward | 14 (41.2%) | 18 (9.7%) |
| - Non-COVID-19 patient care | 7 (20.6%) | 30 (16.2%) |
| - Non-patient care | 4 (11.8%) | 68 (36.8%) |
| **-** Missing | 0 | 6 (3.2%) |
| **Amsterdam UMC, location VUmc** | 20 (58.8%) | 81 (43.8%) |
| **Amsterdam UMC, location AMC** | 14 (41.2%) | 104 (56.2%) |
| **Contact with coworker with COVID-19** | 23 (67.6%) | 92 (49.7%) |
| **Contact with community member with COVID-19** | 9 (26.5%) | 32 (12.4%) |
| **Sex , n women (%)** | 28 (82.4%) | 138 (74.6%) |
| **Age, median (IQR)** | 40 (27-52) | 47 (38-57) |
| **Nose picking, n (%) - any frequency** | **32 (94.1%)** | **153 (82.7%)** |
| - Never | 2 (5.9%) | 32 (17.3%) |
| - Monthly | 9 (26.5%) | 43 (23.2%) |
| - Weekly | 12 (35.3%) | 54 (29.2%) |
| - Daily | 11 (32.4%) | 56 (30.3%) |
| - Every hour | 0 | 0 |
| **Nail biting, n (%) - any frequency** | **10 (29.4%)** | **62 (33.5%)** |
| - Never | 24 (70.6%) | 123 (66.5%) |
| - Monthly | 2 (5.9%) | 26 (14.1%) |
| - Weekly | 6 (17.6%) | 25 (13.5%) |
| - Daily | 2 (5.9%) | 9 (4.9%) |
| - Every hour | 0 | 2 (1.1%) |
| **Wearing glasses, n (%) – weekly/daily** | **17 (50.0%)** | **129 (69.7%)** |
| - Never | 14 (41.2%) | 47 (25.4%) |
| - Monthly | 3 (8.8%) | 9 (4.9%) |
| - Weekly | 1 (2.9%) | 21 (11.4%) |
| - Daily | 16 (47.1%) | 108 (58.4%) |
| **Having a beard, n (%)* - weekly/daily** | **2 (33.3%)** | **16 (30.4%)** |
| - Never | 4 (66.7%) | 30 (65.2%) |
| - Monthly | 0 | 2 (4.3%) |
| - Weekly | 1 (1.7%) | 6 (13.0%) |
| - Daily | 1 (1.7%) | 8 (17.4%) |

Baseline characteristics of participants (n=219) divided into SARS-CoV-2 seropositive and seronegative subgroups. *Males only.
