# Supplementary material for: Why not to pick your nose: Association between nose picking and SARS-CoV-2 incidence, a cohort study in hospital health care workers
Source: PLoS One. 2023 Aug 2;18(8):e0288352. doi: 10.1371/journal.pone.0288352 (PMC10395815; doi:10.1371/journal.pone.0288352)
Supplement: S2 Table — Results of mixed model logistic regression assessing the association between behaviors or physical features and the outcome SARS-CoV-2 infection. ⤉ Nose picking and nail biting were dichotomized into never versus yes (variating from monthly, weekly, daily to every hour). Wearing glasses or having a beard were dichotomized into no (never or monthly) versus yes (variating from weekly to daily) * Males only. Models were corrected for possible confounding of working in different hospitals (location AMC or location VUmc) and departments (intensive care unit (ICU), emergency department (ED), nursing ward, non-COVID-19 patient care, non-patient care) and contact with a COVID-19 infected coworker of community member. (DOCX) [file pone.0288352.s004.docx]

**S2 Table.**

| **Behaviors (dichotomous)⤉** | **Adjusted OR (95% CI)** |
| --- | --- |
| Nose picking | 3.74 (0.98 to 25.06) |
| Nail biting | 0.79 (0.31 to 1.88) |
| Glasses | 0.54 (0.24 to 1.21) |
| Beard***** | 1.27 (0.16-8.00) |

Results of mixed model logistic regression assessing the association between behaviors or physical features and the outcome SARS-CoV-2 infection. **⤉** Nose picking and nail biting were dichotomized into never versus yes (variating from monthly, weekly, daily to every hour). Wearing glasses and having a beard were dichotomized into no (never or monthly) versus yes (variating from weekly to daily) * Males only. Models were corrected for possible confounding of working in different hospitals (location AMC or location VUmc) and departments (intensive care unit (ICU), emergency department (ED), nursing ward, non-COVID-19 patient care, non-patient care) and contact with a COVID-19 infected coworker of community member.
